# Genome-Wide Identification and Analysis of bZIP Transcription Factor Gene Family in Broomcorn Millet (*Panicum miliaceum* L.)

**DOI:** 10.3390/genes16070734

**Published:** 2025-06-24

**Authors:** Peipei An, Tianxiang Liu, Zhijie Shui, Panrong Ren, Shan Duan

**Affiliations:** 1 Gansu Key Laboratory of Conservation and Utilization of Biological Resources and Ecological Restoration in Longdong, School of Agriculture and Bioengineering, Longdong University, Qingyang 745000, China; panrongren@ldxy.edu.cn (P.R.); duanshan@nwafu.edu.com (S.D.); 2 State Key Laboratory of Crop Stress Biology for Arid Areas, College of Agronomy, Northwest A & F University, Yangling 712100, China; ltxiang@nwafu.edu.cn (T.L.); zhijie_shui@nwafu.edu.cn (Z.S.)

**Keywords:** bZIP transcription factor family, *Panicum miliaceum*, genome-wide investigation, phylogenetic analysis, gene structure display, expression analysis during germination, abiotic stress response, abscisic acid (ABA) response

## Abstract

Background: Basic (region) leucine zippers (bZIPs) make up one of the largest families and are some of the most prevalent evolutionarily conserved transcription factors (TFs) in eukaryotic organisms. Plant *bZIP* family members are involved in seed germination, vegetative growth, flower development, light response, and various biotic/abiotic stress response pathways. Nevertheless, a detailed identification and genome-wide analysis of the *bZIP* family genes in broomcorn millet have not been conducted. Methods: In this research, we performed genome-wide identification, phylogenetic analysis, cis-elements analysis, and expression pattern analysis. Results: 144 bZIP transcription factors were identified from the *P. miliaceum* genome and classified into eleven subfamilies using a phylogenetic analysis. Motif and bZIP domain sequence alignment analyses indicated that the members in each subfamily were relatively conserved. Furthermore, a promoter analysis revealed that bZIP transcription factor family genes were responsive to multiple hormones and environmental stresses. Additionally, cis-element MYB binding sites were identified in the promoters of most *PmbZIP* genes. A gene expression analysis showed that 18 *PmbZIP* genes were differentially expressed during seed germination in salt stress, with 7 being significantly downregulated and 11 upregulated, thus suggesting that these *PmbZIP* genes may play an important role in the salt stress response and seed germination. Conclusions: Current research provides valuable information for further functional analyses of the *PmbZIP* gene family and as a reference for future studies on broomcorn millet’s stress response.

## 1. Introduction

Broomcorn millet (*Panicum miliaceum* L.) is one of the plants that was cultivated the earliest in the world and is also known as millet, common millet, or proso millet. It has been cultivated for more than 10,000 years and played significant roles in food security and cultural history in China [1]. Broomcorn millet has a short growing period and is the most water-efficient; has a high salt tolerance and good nutrient resource usage efficiency; and is high in proteins, certain minerals, and antioxidants compared to most other cereals [2,3,4]. Broomcorn millet is an allotetraploid with 36 chromosomes (2n = 4× = 36) [5], and its genome size is approximated to be ~923 Mb [6]. As a C4 photosynthesis crop that is closely related to the bioenergy crop switchgrass (*Panicum virgatum* L.), broomcorn millet has been reported to have the maximum water use efficiency (WUE), which may be due to its slow breathing rate, short generation time (about 60–90 days), and high harvest index [7,8,9]. Progress in molecular biology research on broomcorn millet is slow, and only a simple genetic map and a few genetic markers have been found. In 2019, the millet genome was sequenced, and phylogenetic analysis showed that millet divided into broomcorn millet and foxtail millet ~13.1 million years ago (Mya), while broomcorn millet became an allotetraploid ~5.91 million years ago [10]. One study also identified C4 candidate genes in broomcorn millet, and these genes were found to be spread over all three C4 subtypes, thus suggesting that broomcorn millet coexisted in three different carbon fixation pathways [6]. In the immediate future, broomcorn millet will become a crucial crop that can help in diversifying agriculture and promoting a healthier diet for humans. Therefore, searching for and identifying advantageous genes in millet will facilitate further basic scientific research on molecular breeding processes.

Transcription factors (TFs, also called trans-regulatory factors) are proteins with DNA-binding function, and they bind to specific cis-regulatory elements (cis-elements) and directly regulate the transcription of DNA to mRNA [11]. There are more divergent TF families and more unique DNA-binding domains (DBDs) in plant genomes [12], which also play a vital role in the regulation of growth, development, and environmental response. Structurally, TFs are usually classified by their DNA-binding domains: Basic (region) leucine zipper (bZIP) TFs have a basic region that binds DNA in the N-terminal and a leucine zipper dimerization motif in the C-terminal [13]. The basic DNA-binding region is an invariant N-X7-R/K motif, which has asparagine (N) and basic (R/K) residues with exact spacing; meanwhile, the ZIP domain within an alpha helix consists of heptad repeats of leucine (L) or a related hydrophobic amino acid [14]. Charged amino acids produce attractive and repulsive g-e’ pairs to regulate dimerization specificity and bind to DNA [15].

Among various transcription factor families in plants, basic leucine zipper (bZIP) proteins represent one of the most conserved and extensively studied groups. They are involved in diverse biological processes, particularly in mediating plant responses to abiotic stresses such as drought, salinity, and cold. bZIP transcription factors are well known for their central role in abscisic acid (ABA)-dependent signaling pathways, especially the PP2C–SnRK2–AREB cascade, which regulates key stress-adaptive responses and seed germination [16]. Compared to other transcription factor families (e.g., zinc finger and bHLH), *bZIP* genes have been more directly linked to core hormonal and environmental response mechanisms. To date, many plant bZIP transcription factor families have been identified and characterized extensively, including 78 *AtbZIPs* in *Arabidopsis thaliana* [13,14], 89 *OsbZIPs* in rice (*Oryza sativa*) [17], 187 *TabZIPs* in wheat (*Triticum aestivum*) [18], 85 *SibZIPs* in foxtail millet (*Setaria italica*), 103 *OebZIPs* in olive (*Olea europaea*) [19], 154 *PhebZIPs* in bamboo (*Phyllostachys edulis*) [20], 247 *BnbZIPs* in *Brassica napus* [21], 50 *FvbZIPs* in strawberry (*Fragaria vesca*) [22], 115 *ZlbZIPs* in *Zizania latifolia* [23], and 66 *ItfbZIPs* in sweet potato (*Ipomoea trifida*) [24]. Numerous studies have shown that different subgroups of bZIP TFs are involved in multiple regulation pathways in plants. bZIP TFs in Group A mainly take part in ABA signaling [25,26,27], abiotic stress responses [28,29,30,31], seed germination [32,33], and plant floral transition control [34,35,36]; bZIP TFs in Groups B and K are important regulators of endoplasmic reticulum (ER) stress response [14,37]; those in Groups C and S regulate sucrose signaling and seed storage protein production [38,39,40]; those in Group D are involved in detoxification processes and pathogen defense responses with salicylic acid (SA) defenses against biotrophic pathogens, as well as the defense of hormones jasmonic acid (JA) and ethylene (ET) against necrotrophies [14,41,42,43,44,45,46]; E-members and M-members might control pollen development [47]; F subfamily members control genes that encode for Zn transporters [14,48,49]; those in Groups G and J have been reported to regulate ER–Golgi transport and pathogen defense [50,51]; H-members are implicated in multiple hormone signaling pathways and development during light regulation [52,53,54]; bZIP TFs in Group I participate in osmosensory responses and root bending regulation [55,56]. bZIP target sequences often contain an ACGT core [57], such as G-box (CACGTG), C-box (GACGTC), A-box (TACGTA), etc. However, one investigator also found that some bZIPs can bind non-ACGT sequences [58,59].

Although the function of bZIP TFs has been reported and their identification and analysis have been conducted in Arabidopsis and many species, their roles in broomcorn millet remain largely unknown. In our previous transcriptome analysis of broomcorn millet (*P.miliaceum*) under salt stress [60], we observed that a substantial number of *bZIP* genes were differentially expressed during seed germination. This suggests that bZIP transcription factors may play important regulatory roles in the stress tolerance mechanisms of this highly resilient cereal crop. This study systematically analyzes biological information about bZIP TFs in broomcorn millet, aiming to provide a reference for the identification of various functions.

## 2. Materials and Methods

### 2.1. Genome-Wide Identification and Prediction of Physicochemical Properties

Broomcorn millet genomic sequences, coding region sequences (coding sequence, CDS), and protein sequences were downloaded from NCBI under BioProject number PRJNA431363 (https://www.ncbi.nlm.nih.gov/bioproject/?term=PRJNA431363, accessed on 23 June 2022). We referred to the Pfam number (PF00170) of bZIP transcription factors in the Pfam database (http://pfam.xfam.org/, accessed on 23 June 2022) and used the HMMER program based on a Hidden Markov Model (3.3.2) to search for the candidate bZIP proteins in broomcorn millet. The HMM profile was used to perform an hmmscan search against the *P.miliaceum* protein database with an E-value threshold of 1 × 10^−5^. We used protein sequences with a result greater than “0” as candidate sequences, and then, the candidate gene protein sequence was extracted using TBtools (v1.0692) [61]. Finally, all candidate protein sequences were further detected and identified via CDD (http://www.ncbi.nlm.nih.gov/cdd/, accessed on 22 June 2022) and PFAM, and proteins without bZIP domains were removed. The proteins were named according to the location of the bZIP transcription factor on the chromosome. The theoretical isoelectric point and molecular weight of the bZIP transcription factor proteins in broomcorn millet were calculated using ExPASy—Compute pI/Mw (https://web.expasy.org/compute_pi/, accessed on 25 June 2022).

### 2.2. Phylogeny Analyses and bZIP Domain Amino Acid Sequence Alignment

*A.thaliana* bZIP TF protein sequences were downloaded from the database TAIR (https://www.arabidopsis.org/index.jsp, accessed on 2 July 2022), and *S.italica* bZIP TF protein sequences were downloaded from the database Phytozome v12 (http://phytozome.jgi.doe.gov/pz/portal.html, accessed on 2 July 2022). A bootstrap neighbor-joining (NJ) evolutionary tree was created using MEGA 6.06 (https://www.megasoftware.net/, accessed on 7 July 2022) software with 1000 bootstrap replicates based on the sequence alignments. Additionally, the sequence alignment of the bZIP domain from broomcorn millet was performed using Clustal X 1.8.

### 2.3. Motif and Intron/Exon Gene Structure Analysis

The MEME v5.3.0 online service (http://meme-suite.org/tools/meme, accessed on 13 August 2022) was employed to identify conserved motifs in PmbZIP proteins, using parameters that included a maximum of 12 motifs and an optimal motif width ranging from 6 to 50 amino acids. Afterward, all identified motifs were annotated using InterProScan (http://www.ebi.ac.uk/Tools/pfa/iprscan/, accessed on 16 August 2022), and the gene structure display server program (http://gsds.cbi.pku.edu.cn/, accessed on 30 August 2022) was used to draw the gene structures of *PmbZIPs*.

### 2.4. Promoter Analysis

The 2000 base pairs preceding the initiation codon of each *PmbZIP* gene was obtained. These sequences were analyzed to find cis-elements using the PlantCARE online tool (http://bioinformatics.psb.ugent.be/webtools/plantcare/html/, accessed on 17 October 2022), and the outcomes were visualized with TBtools (v1.0692) [61].

### 2.5. RNA Isolation and bZIP Gene Expression Analysis

The samples of yumi1 and yumi9, which were grown in 0 mM of NaCl (RO water) and 250 mM NaCl, respectively, were collected at 0 h and 3 h, and dew white seeds were harvested after continuous light conditions commenced. (There were three independent biological replicates for each sample). The samples were swiftly frozen with liquid nitrogen and stored at −80 °C. In total, thirty-six samples were employed for RNA-Seq and differential expression analyses.

For transcriptome analysis, total RNA was isolated from seeds using the RNA prep Pure polysaccharide polyphenol plant total RNA extraction kit (DP441) (TIANGEN, Beijing, China). Using 1% agarose gels tested RNA degradation and contamination, and RNA purity was evaluated using a NanoPhotometer^®^ spectrophotometer (IMPLEN, Munich, Germany). RNA concentration was measured using Qubit^®^ RNA Assay Kit in Qubit^®^ 2.0 Flurometer (Life Technologies, Carlsbad, CA, USA). The Agilent Bioanalyzer 2100 system’s RNA Nano 6000 Assay Kit was used to assess RNA integrity (Agilent Technologies, Santa Clara, CA, USA).

A differential expression analysis of two conditions/groups was performed. The DESeq R package (version 1.10.1) was used to analyze differential expression between two conditions or groups. DESeq offers statistical methods to identify differential expression data, utilizing a model based on the negative binomial distribution. The Benjamini and Hochberg method was used to adjust the resulting *p*-values to control the false discovery rate, and differentially expressed genes were those with an adjusted *p*-value under 0.05. The creation of heatmaps and the cluster analysis of *PmbZIPs* were accomplished using TBtools software [61].

## 3. Results

### 3.1. Identification of PmbZIPs in Broomcorn Millet

To identify *bZIP* genes in the complete *P. miliaceum* genome, the Hidden Markov Model (HMM) profile file of the bZIP domain (PF00170) was exploited as a query file for a search across the *P. miliaceum* protein sequence data, and the Pfam and CDD databases were used to confirm the presence of the complete bZIP domain. As shown in Table 1, we identified 144 *PmbZIP* genes in the *P. miliaceum* genome after removing redundant sequences and designated them as *PmbZIP1* to *PmbZIP144* according to their chromosome locus. Moreover, the physical and chemical properties of the 144 *PmbZIPs* were analyzed, such as amino acid (aa) length, molecular weight (MW), and protein isoelectric (PI) points. Chromosomal localization shows that there are 14 *PmbZIPs* in Chr5, which has the most *bZIP* genes, but Chr15, Chr16, and Chr17 only have 2 *PmbZIPs*. Genome sequence analyses were conducted and showed that *PmbZIPs* ranged from 369 base pairs (bp, *PmbZIP100*, *PmbZIP114*) to 12,828 bp (*PmbZIP45*). Protein sequence analyses showed that the *PmbZIPs* ranged from 78 aa (*PmbZIP59*) to 650 aa (PmbZIP51) in length. The predicted MWs ranged from 9.19 kDa (*PmbZIP59*) to 68.3 kDa (PmbZIP51), and the PIs ranged from 4.52 (*PmbZIP102*) to 11.57 (*PmbZIP43*).

### 3.2. Phylogenetic and Sequence Conservation Analysis of PmbZIPs

**Figure 1:**
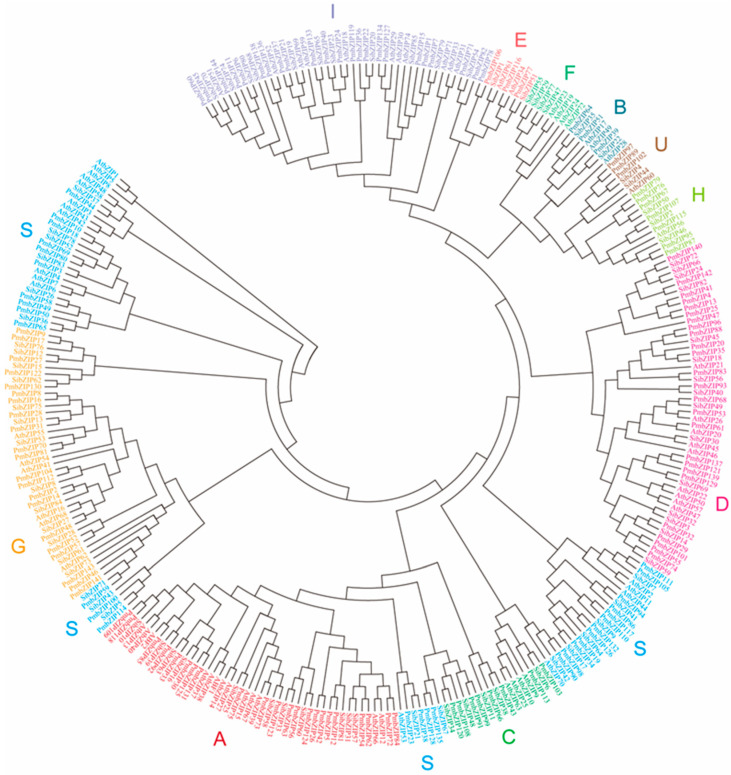
Phylogenetic analysis of bZIP transcription factors in *Arabidopsis thaliana*, *Setaria italica*, and *P. miliaceum*. A maximum likelihood phylogenetic tree was constructed using the full-length bZIP protein sequences from *A. thaliana* (At), *S. italica* (Si), and *P. miliaceum* (Pm). The bZIP proteins were classified into 11 subgroups, each represented by a distinct color. Subgroup names are indicated outside the tree. The grouping was supported by sequence similarity and domain conservation, revealing evolutionary relationships and potential functional conservation among bZIP family members across the three species.

A phylogenetic analysis was performed with all 144 identified PmbZIP proteins, as well as 75 Arabidopsis and 78 foxtail millet bZIP family members (Figure 1 and Figure S1), using the neighbor-joining (NJ) algorithm in MEGA software (MEGA6.06). The 144 *PmbZIP* genes were divided into ten groups (designated as A to E, G, H, I, S, and U) according to the subfamilies classified for Arabidopsis. Based on phylogenetic relationships, Group S contains 29 members and is the largest subfamily, and the smallest subfamilies are Groups B, E, and U, with only 3 members in each. It is interesting to note that no *PmbZIP* member was assigned to Group F. A total of 22 *PmbZIPs* were classified as belonging to Group A, 25 to Group D, 23 to Group I, 21 to Group G, 8 to Group C, and 7 to Group H.

### 3.3. Motif and Structural Analysis of PmbZIPs

**Figure 2:**
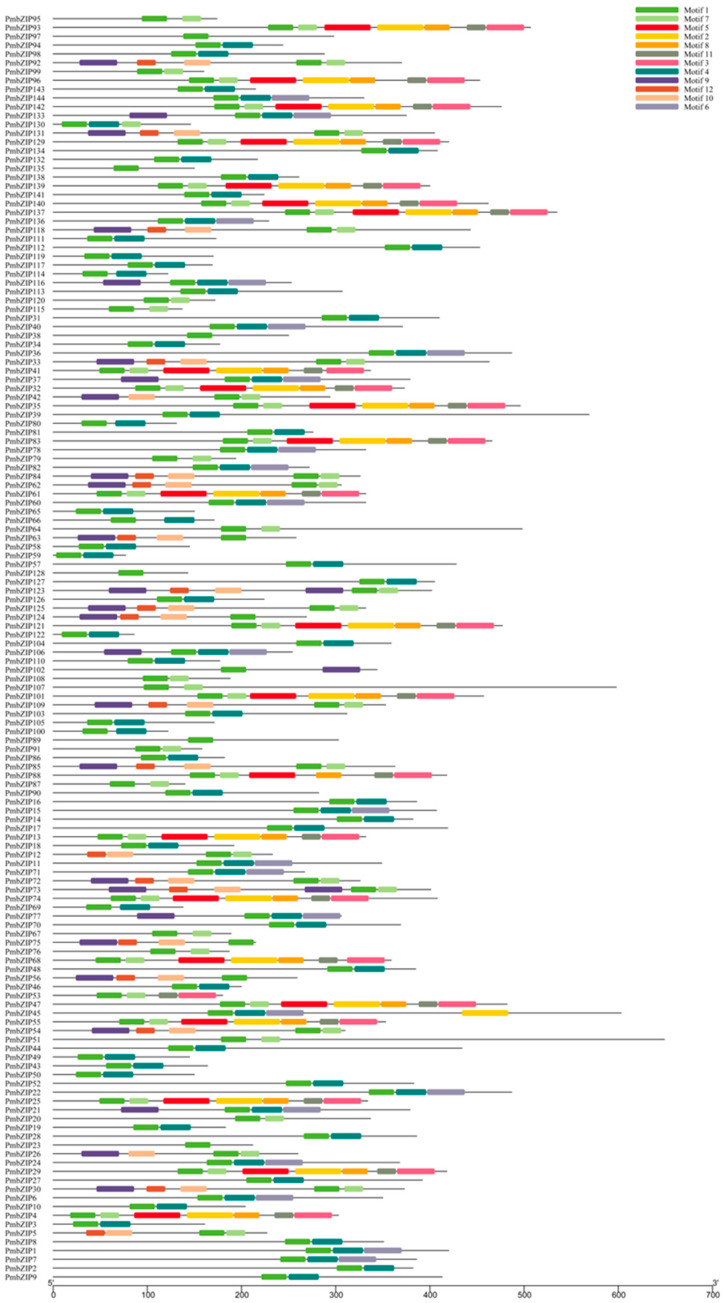
Motif analysis of *P. miliaceum* bZIP proteins. Twelve conserved motifs were identified. Each motif is represented by a colored box with a unique color corresponding to the motif number, as shown in the legend.

To investigate the protein sequence features of *PmbZIPs*, 12 different motifs were identified in *PmbZIPs* (Figure 2), with lengths ranging from 21 to 50 aa. The phylogenetic analysis showed that the same clusters of *PmbZIPs* had similar conserved domain compositions, and obvious differences between different groups were also found. Motif 1, as a “basic region” of the bZIP domain, existed in all *PmbZIPs*. Additionally, motifs 4 and 7, as two different “leucine zippers” of the bZIP domain, existed in Groups B, C, E, G, I, and S and Groups A, D, H, and U, respectively. Specifically, the *PmbZIPs* in Group D contain the most motif types, including motifs 1, 2, 3, 5, 7, 8, and 11. However, only motif 1 was present in *PmbZIP135*, *PmbZIP128*, *PmbZIP38*, and *PmbZIP23* in Group S. This may be the reason why different subfamilies have different functions.

**Figure 3:**
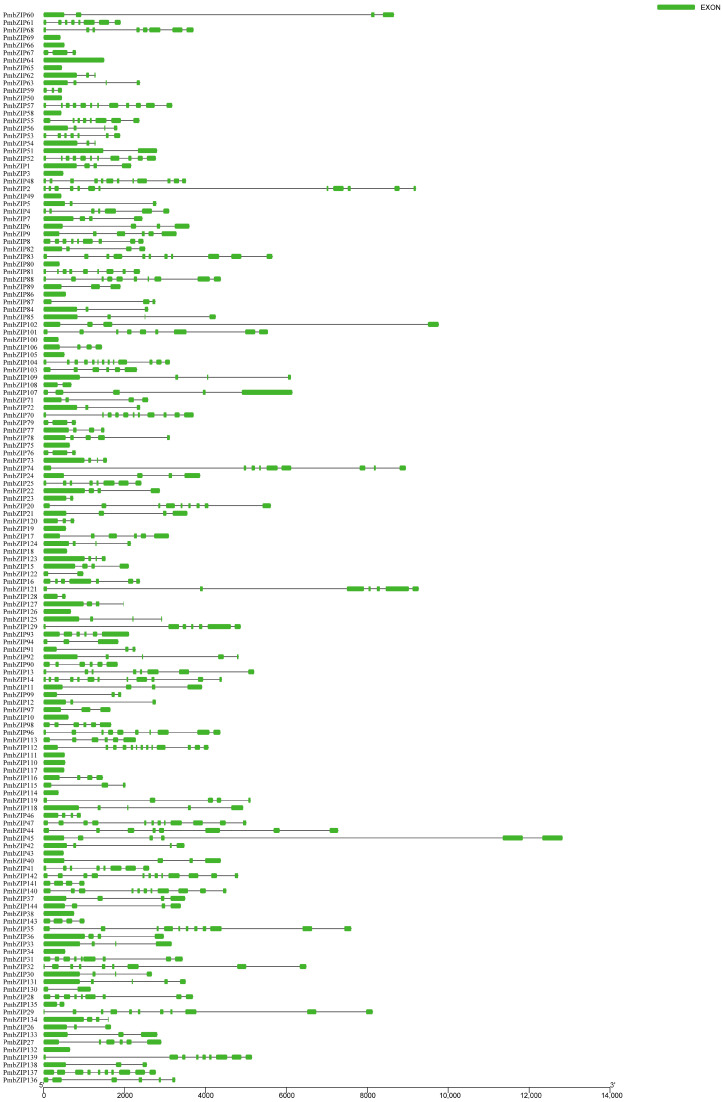
Gene structural analysis of *PmbZIPs*. The exon–intron structures of *bZIP* genes were visualized to compare their gene organization. Green rounded rectangles represent exons, and black solid lines indicate introns.

The structural diversity of the *PmbZIP* family was analyzed in terms of the exon/intron arrangement of the coding sequences. The number of introns in *PmbZIPs* ranged from zero to twelve. The detailed gene structure of *PmbZIPs* is pictured in Figure 3. Twelve introns were identified in *PmbZIP104* and *PmbZIP112*, whereas 24 *PmbZIPs* were identified as intronless. Subgroups G and D generally contain more than seven introns, while subgroup S often has no introns. Most closely related *PmbZIPs* in the same class or subfamily share a similar gene structure in terms of the number of introns.

### 3.4. Promoter Analysis of PmbZIPs

**Figure 4:**
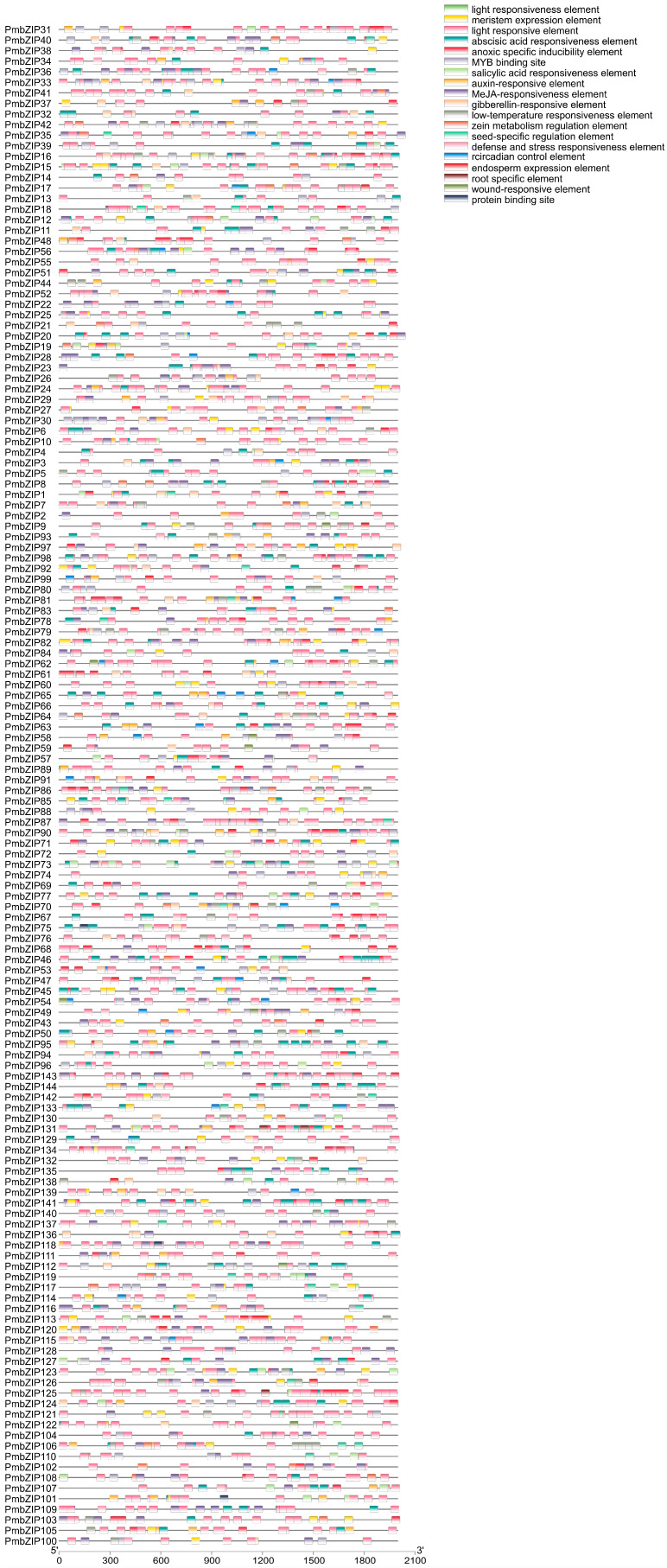
Cis-element analysis of promoter regions of *PmbZIPs*. The cis-acting regulatory elements within the 2000 bp upstream promoter regions of *bZIP* genes were analyzed. Different cis-elements are represented by colored boxes, each corresponding to a specific functional category.

To predict the biological function of *PmbZIPs*, 2000 bp upstream sequences from the translation start sites of *PmbZIPs* were analyzed using the PlantCARE database (Figure 4). The promoter of each *PmbZIP* consists of several cis-acting elements, such as phytohormone-responsive elements, MYB binding sites, light-responsive elements, anoxic-specific inducibility elements, abiotic stress-responsive elements, defense- and stress-responsive elements, seed-specific regulation elements, and root-specific elements. As illustrated in Table 2, a light-responsive element was identified in the promoters of all *PmbZIPs*. An abscisic acid-responsive element, methyl jasmonate (MeJA)-responsive element, and meristem expression element were identified in the promoters of 135, 128, and 99 *PmbZIP* genes, respectively. The promoters of 66 *PmbZIPs* contained an auxin-responsive element, 75 *PmbZIPs* contained a gibberellin-responsive element, and 55 *PmbZIPs* contained a salicylic acid-responsive element. Additionally, an MYB binding site, defense- and stress-responsive elements, a low-temperature-responsive element, and a zein metabolism regulation element were all found in 97, 33, 64, and 46 *PmbZIPs*, respectively. In total, *PmbZIP143* promoters contained 73 (maximum) cis-elements, which included 34 light-responsive elements, 17 abscisic acid-responsive elements, 14 MeJA-responsive elements, 7 anoxic-specific inducibility elements, and 1 meristem expression element. However, *PmbZIP21* promoters only contained 11 cis-elements. These findings demonstrate that *PmbZIPs* might be associated with various transcriptional regulations involving development, hormones, and stress responses.

### 3.5. Expression Analysis of PmbZIPs in Seed Germination Under Salt Stress

**Figure 5:**
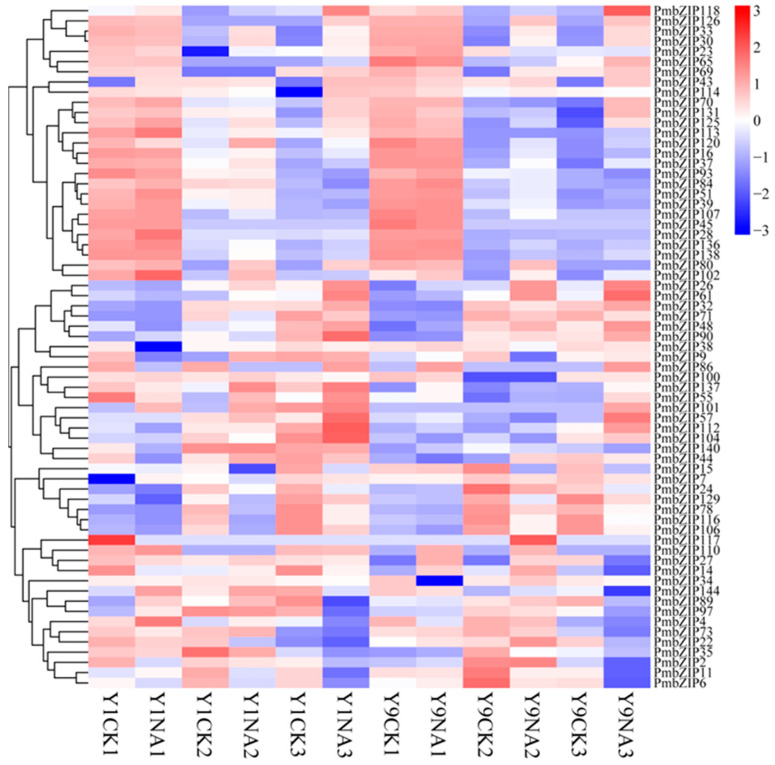
Expression heatmap of 67 differentially expressed *bZIP* genes. Samples were collected from two *P. miliaceum* varieties, Y1 and Y9, under control (CK) and salt treatment (NA) conditions at three time points. Gene expression levels were normalized (log_2_ (FPKM + 1)) and visualized to reveal temporal and treatment-specific expression patterns.

To explore the expression patterns of these millet *bZIP* genes, we used RNA-seq data of yumi1 (Y1) and yumi9 (Y9) under salt stress in the seed germination stage. Based on the millet RNA-seq data, 67 *bZIP* genes were detected in all three sampling stages at the gene level (Figure 5). This suggests that nearly half of *bZIP* genes are broadly expressed during millet germination and development. In addition, the fact that 18 *bZIP* genes have different expression levels suggests that these genes were induced by salt and have a vital function in tolerance responses. The heatmap analysis found that *PmbZIP6*, *PmbZIP11*, *PmbZIP15*, *PmbZIP71*, *PmbZIP78*, *PmbZIP89*, and *PmbZIP97* are downregulated salinity-responsive genes (SRGs). Upregulated SRGs include *PmbZIP26*, *PmbZIP30*, *PmbZIP33*, *PmbZIP65*, *PmbZIP70*, *PmbZIP104*, *PmbZIP107*, *PmbZIP113*, *PmbZIP118*, *PmbZIP125*, and *PmbZIP131*. Interestingly, the differential expression of most SRGs occurs when water is imbibed for 3 h or during radicle protrusion (RAP) in the seed envelope stage, thus indicating that the seeds reinitiate metabolic processes and stress response in this period.

## 4. Discussion

In the immediate future, broomcorn millet will become a crucial crop that can help in diversifying agriculture and promoting a healthier diet for humans. Several plant bZIP transcription factor families have been identified and characterized extensively, which play a vital role in the regulation of growth, development, and environmental response. However, these gene families have not been reported in broomcorn millet. Therefore, searching for and identifying advantageous *bZIP* genes in millet will facilitate further basic scientific research on molecular breeding processes.

### 4.1. Characterization of Broomcorn Millet bZIP Gene Family

The genes involved in genome replication events can evolve into genes with new functions, which play an important role in expanding genome content and diversifying gene functions [62]. Previous research showed that the emergence of the broomcorn millet genome was the result of the ~5.6 MYA hybridization of two closely related genomes. Most Panicum species are polyploids native to the tropical/semi-arid regions of the world. Many gene families in the millet genome are double copies, most of which are retained by the parental species and single-copy genes of the parental species [6]. Since there are 78 *bZIP* genes in the foxtail millet genome, 144 *bZIP* genes were predicted to be in the *P. miliaceum* genome. In addition, many subfamily *bZIP* genes are double copies in broomcorn millet, except those in Groups B, E, F, and U; this is consistent with previous research. After genome duplication, nonfunctionalization (duplicated genes are silenced), subfunctionalization (function is partitioned between the new paralogs), and neofunctionalization (duplicated genes gain new functions) generally take place [63,64,65]. In this study, we found that Group F genes could have been lost or had changed functions during their evolution, thus suggesting that there was extensive gene loss during genome duplication.

A phylogenetic analysis of the *PmbZIP* gene family revealed that subfamilies A, D, and S contain a relatively large number of members that form well-supported and tightly clustered groups, thus suggesting potential functional conservation and lineage-specific expansion. Notably, the S subfamily appears to be divided into four smaller clades, which are interspersed among the members of the G, A, and C subfamilies. This branching pattern suggests that during evolution, some ancestral S-type bZIP members may have undergone functional divergence and structural differentiation, thus giving rise to new regulatory subgroups such as G, A, and C. These findings support the hypothesis that bZIP subfamily diversification has been driven by both sequence divergence and the acquisition of specialized regulatory roles in different stress and developmental contexts.

Among these subfamilies, Group A *bZIP* genes stand out due to their well-established roles in abscisic acid (ABA)-mediated stress responses. In our study, *PmbZIP30* (*PmABI5*) and *PmbZIP131*, both belonging to Group A, were significantly induced by ABA during seed development in broomcorn millet [60]. This is consistent with findings in Arabidopsis and rice, where homologous Group A *bZIPs* (e.g., ABI5) act as core components in the ABA signaling pathway. Furthermore, cross-species evidence supports the involvement of Group A *bZIPs* in the conserved PP2C–SnRK2–AREB signaling module, which regulates plant responses to drought and other abiotic stresses. The promoter architecture and expression dynamics of Group A *PmbZIP* genes in this study reinforce the notion that their functional roles in ABA-dependent stress signaling are evolutionarily conserved. These results not only provide insight into the diversification and conservation of bZIP transcription factors in broomcorn millet but also highlight the adaptive significance of Group A members in stress tolerance.

### 4.2. Structural Analysis of PmbZIPs

The detailed understanding of the functional domain of *A. thaliana* and *S. italica* bZIPs enabled us to analyze similar domains within the broomcorn millet *bZIP* gene family. In this research, all 144 *PmbZIP* proteins contain the necessary basic domain, which provides the structural basis for their conserved function. Moreover, different subgroups have different ZIP motifs, which makes their functions differ. Groups A, D, H, and U have type I ZIP domains (motif 7, K-L-X7-R), and the main function of these subfamily genes is the regulation of biological and abiotic stresses. However, Groups B, C, E, G, I, and S have type II ZIP domains (motif 4, V-L-X8-R) that are involved in carbohydrate biosynthesis, post-transcriptional inhibition, development, and hormone synthesis. It has been reported that intron retention regulates protein isoform production, RNA stability and translation efficiency, and the rapid induction of expression via the post-transcriptional splicing of retained introns [66]. An analysis of the bZIP gene structure revealed that most intronless PmbZIPs occurred in Groups S and C, and a similar observation was reported for banana (*Musa spp.* L.) and switchgrass (*P. virgatum* L.) [67,68]. The *PmbZIPs* of subgroups A, C, and I, with relatively fewer introns, were associated with stress responses. This conclusion aligns with the results of the present and past studies [60].

### 4.3. Cis-Element Analysis in the Promoters of PmbZIPs

The cis-elements of different transcription factors have different functions. The number and form of cis-elements in promoter regions could play an essential role in the regulation of gene expression. The results illustrate that abiotic stress-related cis-elements, including abscisic acid-responsive elements, anoxic-specific inducibility elements, low-temperature-responsive elements, MeJA-responsive elements, MYB binding sites, salicylic acid-responsive elements, and wound-responsive elements, are major regulatory elements in the *PmbZIP* promoters activated by ABA, NaCl, or other forms of abiotic stress. In addition, many development-related cis-elements were also found, such as auxin-responsive elements, endosperm expression elements, gibberellin-responsive elements, root-specific elements, seed-specific regulation elements, and meristem expression elements. These findings suggest that light-responsive elements, abscisic acid-responsive elements, and MeJA-responsive elements play a vital role in transcriptional regulation in broomcorn millet. *PmbZIP* promoters present a lot of stress-responsive cis-elements and hormone response cis-elements, which indicates their potential roles in the response to stress and pathogen infections. Consequently, *PmbZIPs* are often taken as candidate genes to understand the responses to biotic stresses and plant development.

### 4.4. PmbZIP Involvement in Development and Stress Response

Previous reports have revealed that bZIP TFs function in many stress responses and development by regulating diverse biochemical and physiological pathways [17,69,70,71]. bZIP transcription factors possess different characteristics in different species. The overexpression of *TabZIP15* in wheat can enhance root length and fresh weight during salt stress, thus suggesting that the *TabZIP15* gene is involved in the regulation of wheat salt stress tolerance [72]. The homozygous T-DNA insertional mutants *Osabf1-1* and *Osabf1-2* are more sensitive in response to drought and salinity treatments than wild-type plants, and the *OsNAC*, *OsLEA3*, and *OsABA45* genes are significantly suppressed in *Osabf1* mutants. Hence, *OsABF1* likely plays a positive role as an ABA-responsive transcription factor in abiotic stress signaling [26]. We incorporated and highlighted experimental data from our previous work, wherein *PmbZIP30* was overexpressed in rice. The transgenic rice lines exhibited significantly shorter seed germination times compared to the wild-type, thus indicating a positive regulatory role of *PmbZIP30* in seed germination and stress response. This functional validation supports the biological relevance of our bioinformatic findings (Figure S2). The overexpression of *StbZIP65* in Arabidopsis enhanced salt tolerance [73]. However, some research shows that *GmbZIP19* expression is significantly induced by ABA (abscisic acid), JA (jasmonic acid), and SA (salicylic acid) but is reduced under salt and drought stress conditions, thus suggesting that *GmbZIP19* is a positive regulator of pathogen resistance and a negative regulator of salt and drought stress tolerance [74]. *TabZIP6* can bind to the promoters of *CBFs* and decrease the expression of downstream *COR* genes in *TabZIP6*-overexpressing Arabidopsis seedlings; therefore, *TabZIP6* is a negative regulator in the cold stress response [75].

In this study, a total of 144 *PmbZIP* genes were identified in the *P. miliaceum* genome and classified into eleven subfamilies based on phylogenetic relationships. Conserved motif and domain structure analyses revealed that the members within each subfamily exhibit high sequence conservation. The promoter analysis indicated that *PmbZIP* genes may be involved in multiple hormone signaling pathways and environmental stress responses, as reflected by the presence of diverse cis-acting regulatory elements, including MYB binding sites. The transcriptome analysis further showed that 18 *PmbZIP* genes were differentially expressed during seed germination under salt stress, thus suggesting their potential regulatory roles in abiotic stress adaptation. These findings provide a valuable resource for understanding the functional roles of bZIP transcription factors in broomcorn millet and lay the foundation for future stress resilience breeding and gene function studies.

## Figures and Tables

**Table 1:** Identification and summary of information on *Panicum miliaceum* bZIP transcription factors (*PmbZIPs*) in broomcorn millet.

Gene ID	Protein Number	Gene Number	Chr	Gene Location	Gene Length/bp	CDS/nt	Amino Acids	PI	Molecular Weight/u
*PmbZIP1*	RLN41431.1	PM01G00370	1	454801–457374	2574	1263	421	5.71	44,828.00
*PmbZIP2*	RLN41707.1	PM01G08790	1	7046270–7059039	12,770	1149	383	9.13	40,969.23
*PmbZIP3*	RLN40362.1	PM01G12600	1	10097025–10098402	1378	486	162	10.55	17,692.55
*PmbZIP4*	RLN40088.1	PM01G13370	1	10863218–10866321	3104	912	304	9.45	34,047.74
*PmbZIP5*	RLN40734.1	PM01G13620	1	11058035–11060820	2786	684	228	9.84	24,396.40
*PmbZIP6*	RLN39254.1	PM01G14300	1	11670497–11674442	3946	1053	351	6.23	37,904.15
*PmbZIP7*	RLN41556.1	PM01G19740	1	16954898–16957707	2810	1161	387	7.23	41,543.20
*PmbZIP8*	RLN41102.1	PM01G22630	1	19957313–19960138	2826	1056	352	6.88	37,179.95
*PmbZIP9*	RLN42614.1	PM01G23610	1	20646169–20649807	3639	1242	414	5.06	42,598.17
*PmbZIP10*	RLN40049.1	PM01G26880	1	22949190–22950304	1115	615	205	10.18	22,321.56
*PmbZIP11*	RLN19317.1	PM02G26900	2	39030059–39034459	4401	1050	350	6.28	37,679.93
*PmbZIP12*	RLN19126.1	PM02G27550	2	39595051–39597823	2773	702	234	10.00	24,691.66
*PmbZIP13*	RLN18095.1	PM02G27800	2	39766627–39771830	5204	999	333	7.04	37,066.87
*PmbZIP14*	RLN17017.1	PM02G32070	2	43256944–43264901	7958	1149	383	9.20	40,881.04
*PmbZIP15*	RLN15790.1	PM02G38970	2	48295652–48298282	2631	1224	408	5.60	43,833.94
*PmbZIP16*	RLN15762.1	PM02G44000	2	51665787–51668165	2379	1161	387	6.10	41,310.03
*PmbZIP17*	RLN17495.1	PM02G44930	2	52341406–52345126	3721	1260	420	4.97	43,404.97
*PmbZIP18*	RLN18647.1	PM02G46570	2	53532299–53532877	579	579	193	10.48	21,390.51
*PmbZIP19*	RLN33487.1	PM03G00660	3	678937–679488	552	552	184	7.21	20,564.99
*PmbZIP20*	RLN33484.1	PM03G01530	3	1468835–1474450	5616	1014	338	9.88	36,231.59
*PmbZIP21*	RLN33244.1	PM03G03170	3	2666045–2670259	4215	1140	380	6.22	40,613.30
*PmbZIP22*	RLN32939.1	PM03G03540	3	2944837–2947710	2874	1464	488	9.20	51,669.75
*PmbZIP23*	RLN34898.1	PM03G04810	3	3889634–3890848	1215	639	213	6.84	22,223.95
*PmbZIP24*	RLN35613.1	PM03G16340	3	12865234–12869532	4299	1107	369	6.61	39,460.24
*PmbZIP25*	RLN33050.1	PM03G16790	3	13287052–13290598	3547	1005	335	8.90	37,308.18
*PmbZIP26*	RLN35551.1	PM03G16900	3	13342155–13343818	1664	783	261	10.02	28,735.61
*PmbZIP27*	RLN35711.1	PM03G21650	3	18768221–18771281	3061	1179	393	5.01	42,558.22
*PmbZIP28*	RLN33934.1	PM03G24730	3	22107028–22110718	3691	1161	387	6.61	39,600.43
*PmbZIP29*	RLN35629.1	PM03G29190	3	35167059–35175866	8808	1257	419	8.44	46,181.77
*PmbZIP30*	RLN35964.1	PM03G36770	3	57531413–57534679	3267	1122	374	5.10	39,436.23
*PmbZIP31*	RLM84895.1	PM04G07270	4	6860114–6863550	3437	1233	411	7.18	42,128.33
*PmbZIP32*	RLM86983.1	PM04G16890	4	29725581–29732074	6494	1122	374	9.54	41,241.35
*PmbZIP33*	RLM85912.1	PM04G17970	4	31027628–31030792	3165	1392	464	9.15	49,993.48
*PmbZIP34*	RLM85616.1	PM04G18960	4	31857501–31858542	1042	534	178	8.95	20,269.87
*PmbZIP35*	RLM87231.1	PM04G19820	4	32472417–32480988	8572	1491	497	7.51	55,166.25
*PmbZIP36*	RLM85816.1	PM04G21680	4	33883522–33886493	2972	1464	488	9.53	51,605.73
*PmbZIP37*	RLM86211.1	PM04G22100	4	34132892–34136810	3919	1140	380	6.38	40,629.39
*PmbZIP38*	RLM85470.1	PM04G22810	4	34716923–34718185	1263	753	251	7.83	26,101.09
*PmbZIP39*	RLM87366.1	PM04G29540	4	39691716–39694242	2527	1491	497	5.86	60,644.54
*PmbZIP40*	RLM85147.1	PM04G34030	4	42747464–42752113	4650	1116	372	6.27	39,659.45
*PmbZIP41*	RLM86171.1	PM04G34490	4	43064867–43067475	2609	1014	338	8.90	37,565.47
*PmbZIP42*	RLM87099.1	PM04G34620	4	43157454–43160933	3480	885	295	9.59	32,209.76
*PmbZIP43*	RLN30372.1	PM05G00470	5	481032–481791	760	495	165	11.57	17,411.98
*PmbZIP44*	RLN29606.1	PM05G02460	5	2429710–2436988	7279	1305	435	5.93	47,002.20
*PmbZIP45*	RLN28633.1	PM05G04900	5	4731398–4744225	12,828	1812	604	4.88	65,083.35
*PmbZIP46*	RLN27945.1	PM05G05230	5	5015435–5016619	1185	603	201	9.19	21,729.48
*PmbZIP47*	RLN28345.1	PM05G05380	5	5101092–5106900	5809	1449	483	6.25	52,474.70
*PmbZIP48*	RLN27488.1	PM05G08030	5	7121364–7128050	6687	1158	386	7.17	40,824.69
*PmbZIP49*	RLN29878.1	PM05G11380	5	10166559–10169754	3196	438	146	9.49	16,063.02
*PmbZIP50*	RLN30397.1	PM05G14850	5	13174076–13175991	1916	453	151	6.32	16,585.54
*PmbZIP51*	RLN29599.1	PM05G25250	5	46340685–46343488	2804	1950	650	8.15	68,282.36
*PmbZIP52*	RLN31068.1	PM05G29120	5	49501420–49504193	2774	1152	384	9.07	41,007.77
*PmbZIP53*	RLN28247.1	PM05G33670	5	53089874–53091765	1892	543	181	9.75	20,078.72
*PmbZIP54*	RLN28944.1	PM05G33910	5	53238531–53239814	1284	933	311	6.47	33,380.34
*PmbZIP55*	RLN28901.1	PM05G36440	5	55255118–55257485	2368	1062	354	6.68	39,751.11
*PmbZIP56*	RLN27558.1	PM05G36770	5	55582519–55584338	1820	780	260	9.55	26,952.16
*PmbZIP57*	RLN01174.1	PM06G02700	6	1946640–1949819	3180	1287	429	9.30	45,780.45
*PmbZIP58*	RLN00773.1	PM06G08160	6	5838864–5840465	1602	438	146	9.93	15,983.02
*PmbZIP59*	RLN01167.1	PM06G14080	6	10158766–10159216	451	234	78	10.63	9190.60
*PmbZIP60*	RLM98944.1	PM06G14410	6	10441629–10450559	8931	999	333	6.26	35,600.39
*PmbZIP61*	RLM98085.1	PM06G17810	6	12976476–12980172	3697	999	333	6.55	36,500.10
*PmbZIP62*	RLM98033.1	PM06G17990	6	13102719–13104001	1283	921	307	6.46	32,832.58
*PmbZIP63*	RLN00565.1	PM06G20460	6	14965993–14968992	3000	777	259	9.73	26,937.16
*PmbZIP64*	RLM99485.1	PM06G21690	6	16055800–16057296	1497	1497	499	7.03	52,598.69
*PmbZIP65*	RLM99017.1	PM06G30890	6	39341652–39342104	453	453	151	6.85	16,674.63
*PmbZIP66*	RLM99272.1	PM06G35160	6	43290439–43290954	516	516	172	11.51	18,029.63
*PmbZIP67*	RLN24624.1	PM07G00440	7	317276–318073	798	570	190	9.98	20,307.29
*PmbZIP68*	RLN25705.1	PM07G06850	7	6745210–6748914	3705	1080	360	7.69	40,074.24
*PmbZIP69*	RLN24094.1	PM07G10230	7	8252890–8253306	417	417	139	7.65	15,279.28
*PmbZIP70*	RLN24562.1	PM07G17750	7	36088695–36092402	3708	1110	370	9.19	39,245.04
*PmbZIP71*	RLN22304.1	PM07G23470	7	41273483–41276065	2583	804	268	5.93	28,650.69
*PmbZIP72*	RLN22621.1	PM07G25750	7	43086731–43089115	2385	981	327	7.18	35,585.99
*PmbZIP73*	RLN22673.1	PM07G29370	7	45806587–45808150	1564	1206	402	6.29	42,396.98
*PmbZIP74*	RLN23257.1	PM07G29380	7	45812241–45821193	8953	1227	409	6.90	46,071.37
*PmbZIP75*	RLN24712.1	PM07G29810	7	46066329–46066976	648	648	216	9.75	21,645.44
*PmbZIP76*	RLN25012.1	PM07G36760	7	51457907–51459054	1148	564	188	10.01	20,446.76
*PmbZIP77*	RLN24441.1	PM07G38580	7	53023815–53026143	2329	921	307	6.62	32,963.72
*PmbZIP78*	RLM93109.1	PM08G01760	8	1448785–1451902	3118	999	333	6.00	36,038.19
*PmbZIP79*	RLM93604.1	PM08G03930	8	3296164–3296959	796	585	195	9.94	20,689.71
*PmbZIP80*	RLM91692.1	PM08G15750	8	30944899–30945294	396	396	132	8.40	14,577.57
*PmbZIP81*	RLM92180.1	PM08G20990	8	36451978–36454357	2380	831	277	9.31	30,542.73
*PmbZIP82*	RLM93703.1	PM08G25890	8	40248925–40252095	3171	819	273	6.01	28,971.03
*PmbZIP83*	RLM92419.1	PM08G28810	8	42140458–42146113	5656	1401	467	6.34	50,908.00
*PmbZIP84*	RLM94116.1	PM08G29030	8	42303295–42305878	2584	981	327	7.15	35,542.86
*PmbZIP85*	RLN12842.1	PM09G06500	9	5334910–5339163	4254	1092	364	8.70	39,102.69
*PmbZIP86*	RLN12841.1	PM09G12140	9	21889497–21890045	549	549	183	9.67	20,244.55
*PmbZIP87*	RLN13025.1	PM09G17690	9	43522067–43524827	2761	423	141	10.16	15,149.82
*PmbZIP88*	RLN12905.1	PM09G18590	9	44792601–44796981	4381	1257	419	9.11	44,598.29
*PmbZIP89*	RLN12453.1	PM09G18780	9	45041072–45044603	3532	912	304	4.83	32,658.00
*PmbZIP90*	RLN13126.1	PM09G20800	9	47002029–47003859	1831	849	283	6.24	30,013.48
*PmbZIP91*	RLN12623.1	PM09G24100	9	50246233–50248502	2270	477	159	9.00	17,365.70
*PmbZIP92*	RLM55632.1	PM10G06050	10	4576359–4581176	4818	1113	371	9.00	40,165.13
*PmbZIP93*	RLM54848.1	PM10G06130	10	4623806–4625917	2112	1524	508	9.16	55,261.12
*PmbZIP94*	RLM55196.1	PM10G10750	10	10699576–10701422	1847	735	245	10.88	27,271.78
*PmbZIP95*	RLM54794.1	PM10G15500	10	26027305–26029674	2370	525	175	9.75	18,810.58
*PmbZIP96*	RLM56218.1	PM10G16250	10	26722529–26726897	4369	1362	454	9.13	48,594.99
*PmbZIP97*	RLM55027.1	PM10G16410	10	26862899–26864549	1651	897	299	5.43	32,538.26
*PmbZIP98*	RLM55428.1	PM10G18250	10	28349450–28351117	1668	867	289	6.07	30,673.21
*PmbZIP99*	RLM56082.1	PM10G21570	10	31094548–31096463	1916	483	161	8.62	17,579.92
*PmbZIP100*	RLN09938.1	PM11G00290	11	452913–453281	369	369	123	8.97	13,810.34
*PmbZIP101*	RLN08312.1	PM11G02090	11	2470988–2476530	5543	1374	458	8.92	49,484.85
*PmbZIP102*	RLN07652.1	PM11G02220	11	2606668–2616433	9766	1035	345	4.52	35,333.37
*PmbZIP103*	RLN09078.1	PM11G02900	11	3245503–3248289	2787	939	313	5.42	33,095.76
*PmbZIP104*	RLN07125.1	PM11G13460	11	25503439–25506558	3120	1080	360	5.79	38,511.02
*PmbZIP105*	RLN09230.1	PM11G13790	11	25784422–25786801	2380	516	172	6.29	17,688.93
*PmbZIP106*	RLN07329.1	PM11G19430	11	38531228–38532874	1647	765	255	5.78	27,050.34
*PmbZIP107*	RLN08025.1	PM11G20550	11	40200637–40206785	6149	1797	599	9.76	65,299.21
*PmbZIP108*	RLN07962.1	PM11G20760	11	40430132–40431310	1179	567	189	9.02	20,553.29
*PmbZIP109*	RLN09062.1	PM11G24760	11	43626713–43632822	6110	1062	354	6.48	37,768.04
*PmbZIP110*	RLN07622.1	PM11G27250	11	46387650–46388183	534	534	178	8.65	20,643.10
*PmbZIP111*	RLM78368.1	PM12G01810	12	1318893–1319414	522	522	174	6.83	17,739.04
*PmbZIP112*	RLM78875.1	PM12G02550	12	1757193–1761266	4074	1362	454	8.20	47,603.58
*PmbZIP113*	RLM80422.1	PM12G04340	12	3257399–3260279	2881	924	308	5.20	32,615.15
*PmbZIP114*	RLM79681.1	PM12G05380	12	4052057–4052425	369	369	123	9.35	13,871.48
*PmbZIP115*	RLM80771.1	PM12G06150	12	4748641–4750662	2022	414	138	10.11	15,166.99
*PmbZIP116*	RLM79948.1	PM12G08110	12	6857609–6859787	2179	762	254	5.78	26,937.22
*PmbZIP117*	RLM79671.1	PM12G27010	12	38519464–38520329	866	510	170	7.93	19,646.96
*PmbZIP118*	RLM78209.1	PM12G28080	12	39453559–39458489	4931	1332	444	9.41	47,492.18
*PmbZIP119*	RLM79651.1	PM12G29310	12	40484976–40490089	5114	513	171	4.93	18,799.88
*PmbZIP120*	RLM80583.1	PM12G31970	12	42325452–42326207	756	519	173	9.00	18,729.10
*PmbZIP121*	RLN05398.1	PM13G04720	13	5033013–5042282	9270	1434	478	7.24	51,950.15
*PmbZIP122*	RLN05595.1	PM13G09960	13	25084063–25085042	980	261	87	9.65	9705.76
*PmbZIP123*	RLN04073.1	PM13G14010	13	35015891–35017421	1531	1209	403	6.37	42,408.97
*PmbZIP124*	RLN05004.1	PM13G14470	13	35292857–35295671	2815	810	270	9.25	28,271.05
*PmbZIP125*	RLN04831.1	PM13G19990	13	41033378–41036302	2925	999	333	5.76	34,879.32
*PmbZIP126*	RLN04579.1	PM13G20830	13	41770762–41771759	998	675	225	8.54	24,211.82
*PmbZIP127*	RLN03801.1	PM13G24510	13	45184706–45186681	1976	1218	406	6.61	43,025.01
*PmbZIP128*	RLN03549.1	PM13G25090	13	45582601–45583142	542	432	144	10.02	15,452.52
*PmbZIP129*	RLM61107.1	PM14G04080	14	3139207–3146477	7271	1263	421	6.53	45,595.62
*PmbZIP130*	RLM60470.1	PM14G09560	14	20687606–20688770	1165	441	147	9.34	16,666.93
*PmbZIP131*	RLM60601.1	PM14G16770	14	29385478–29388989	3512	1218	406	5.52	42,836.29
*PmbZIP132*	RLM61427.1	PM14G17480	14	30010114–30011044	931	654	218	8.82	23,453.05
*PmbZIP133*	RLM60401.1	PM14G19800	14	31888088–31890896	2809	1128	376	6.32	39,473.30
*PmbZIP134*	RLM61133.1	PM14G19990	14	32037397–32038999	1603	1227	409	6.43	43,304.39
*PmbZIP135*	RLM61596.1	PM14G21450	14	33079164–33079675	512	453	151	9.55	16,265.44
*PmbZIP136*	RLM74471.1	PM15G12970	15	28520231–28523482	3252	690	230	9.83	24,604.94
*PmbZIP137*	RLM74357.1	PM15G25820	15	39118732–39121503	2772	1608	536	7.97	58,416.77
*PmbZIP138*	RLM65300.1	PM16G12130	16	25119044–25121594	2551	786	262	5.10	27,207.24
*PmbZIP139*	RLM66084.1	PM16G21310	16	31781217–31790325	9109	1203	401	6.18	44,727.90
*PmbZIP140*	RLM70069.1	PM17G01920	17	1423232–1427747	4516	1389	463	6.25	50,766.87
*PmbZIP141*	RLM69114.1	PM17G02070	17	1594724–1595732	1009	675	225	8.98	24,596.61
*PmbZIP142*	RLM59076.1	PM18G00820	18	648118–653777	5660	1431	477	6.09	52,218.33
*PmbZIP143*	RLM57820.1	PM18G00930	18	761889–762898	1010	648	216	8.61	23,467.31
*PmbZIP144*	RLM58717.1	PM18G01220	18	1078921–1083128	4208	993	331	6.37	35,578.56

**Table 2:** A summary of cis-elements in the *PmbZIPs* promoter.

Cis-Acting Element	Element Number	Gene Number
Abscisic acid responsiveness element	618	135
Anoxic specific inducibility element	220	90
Auxin-responsive element	95	66
Defense and stress responsiveness element	39	33
Endosperm expression element	29	25
Gibberellin-responsive element	108	75
Light responsive element	1655	144
Low-temperature responsiveness element	95	64
MeJA-responsiveness element	752	128
Meristem expression element	154	99
MYB binding site	175	97
Protein binding site	4	4
Rcircadian control element	40	30
Root specific element	7	5
Salicylic acid responsiveness element	72	50
Seed-specific regulation element	30	26
Wound-responsive element	8	8
Zein metabolism regulation element	61	46

## Data Availability

The original contributions presented in this study are included in the article/Supplementary Materials. Further inquiries can be directed to the corresponding author.

## Supplementary Materials

The following supporting information can be downloaded at https://www.mdpi.com/article/10.3390/genes16070734/s1. Figure S1: Sequence conservation analysis of *PmbZIPs*. Figure S2: Overexpression of PmbZIP30 in rice accelerates seed germination.

## References

[1] Lu H., Zhang J., Liu K.-B., Wu N., Li Y., Zhou K., Ye M., Zhang T., Zhang H., Yang X. (2009). Earliest domestication of common millet (*Panicum miliaceum*) in East Asia extended to 10,000 years ago. Proc. Natl. Acad. Sci. USA.

[2] Yue H., Wang M., Liu S., Du X., Song W., Nie X. (2016). Transcriptome-wide identification and expression profiles of the WRKY transcription factor family in Broomcorn millet (*Panicum miliaceum* L.). BMC Genom..

[3] Rajput S.G., Santra D.K., Schnable J.J.M.B. (2016). Mapping QTLs for morpho-agronomic traits in proso millet (*Panicum miliaceum* L.). Mol. Breed..

[4] Hunt H.V., Badakshi F., Romanova O., Howe C.J., Jones M.K., Heslop-Harrison J.S. (2014). Reticulate evolution in Panicum (Poaceae): The origin of tetraploid broomcorn millet, *P. miliaceum*. J. Exp. Bot..

[5] Wang H., Wang J., Chen C., Chen L., Li M., Qin H., Tian X., Hou S., Yang X., Jian J. (2024). A complete reference genome of broomcorn millet. Sci. Data.

[6] Zou C., Li L., Miki D., Li D., Tang Q., Xiao L., Rajput S., Deng P., Peng L., Jia W. (2019). The genome of broomcorn millet. Nat. Commun..

[7] Baltensperger D.D. (1996). Foxtail and Proso Millet. Progress in New Crops.

[8] Washburn J.D., Schnable J.C., Davidse G., Pires J.C. (2015). Phylogeny and photosynthesis of the grass tribe Paniceae. Am. J. Bot..

[9] Cedric H., Matanguihan J.B., D’Alpoim Guedes J., Ganjyal G.M., Whiteman M.R., Kidwell K.K., Murphy K.M. (2016). Proso Millet (*Panicum miliaceum* L.) and Its Potential for Cultivation in the Pacific Northwest, U.S.: A Review. Front. Plant Sci..

[10] Shi J., Ma X., Zhang J., Zhou Y., Liu M., Huang L., Sun S., Zhang X., Gao X., Zhan W. (2019). Chromosome conformation capture resolved near complete genome assembly of broomcorn millet. Nat. Commun..

[11] Lai X., Chahtane H., Martin-Arevalillo R., Zubieta C., Parcy F. (2020). Contrasted evolutionary trajectories of plant transcription factors. Curr. Opin. Plant Biol..

[12] de Mendoza A., Sebé-Pedrós A., Šestak M.S., Matejcic M., Torruella G., Domazet-Loso T., Ruiz-Trillo I. (2013). Transcription factor evolution in eukaryotes and the assembly of the regulatory toolkit in multicellular lineages. Proc. Natl. Acad. Sci. USA.

[13] Jakoby M., Weisshaar B., Dröge-Laser W., Vicente-Carbajosa J., Tiedemann J., Kroj T., Parcy F. (2002). bZIP transcription factors in Arabidopsis. Trends Plant Sci..

[14] Droge-Laser W., Snoek B.L., Snel B., Weiste C. (2018). The Arabidopsis bZIP transcription factor family-an update. Curr. Opin. Plant Biol..

[15] Deppmann C.D., Acharya A., Rishi V., Wobbes B., Smeekens S., Taparowsky E.J., Vinson C. (2004). Dimerization specificity of all 67 B-ZIP motifs in *Arabidopsis thaliana*: A comparison to Homo sapiens B-ZIP motifs. Nucleic Acids Res..

[16] Mathura S.R., Sutton F., Bowrin V. (2023). Characterization and expression analysis of SnRK2, PYL, and ABF/AREB/ABI5 gene families in sweet potato. PLoS ONE.

[17] Nijhawan A., Jain M., Tyagi A.K., Khurana J.P. (2008). Genomic survey and gene expression analysis of the basic leucine zipper transcription factor family in rice. Plant Physiol..

[18] Li X., Gao S., Tang Y., Li L., Zhang F., Feng B., Fang Z., Ma L., Zhao C. (2015). Genome-wide identification and evolutionary analyses of bZIP transcription factors in wheat and its relatives and expression profiles of anther development related *TabZIP* genes. BMC Genom..

[19] Rong S., Wu Z., Cheng Z., Zhang S., Liu H., Huang Q. (2020). Genome-Wide Identification, Evolutionary Patterns, and Expression Analysis of *bZIP* Gene Family in Olive (*Olea europaea* L.). Genes.

[20] Pan F., Wu M., Hu W., Liu R., Yan H., Xiang Y. (2019). Genome-Wide Identification and Expression Analyses of the bZIP Transcription Factor Genes in moso bamboo (*Phyllostachys edulis*). Int. J. Mol. Sci..

[21] Zhou Y., Xu D., Jia L., Huang X., Ma G., Wang S., Zhu M., Zhang A., Guan M., Lu K. (2017). Genome-Wide Identification and Structural Analysis of bZIP Transcription Factor Genes in *Brassica napus*. Genes.

[22] Wang X.L., Chen X., Yang T.B., Cheng Q., Cheng Z.M. (2017). Genome-Wide Identification of bZIP Family Genes Involved in Drought and Heat Stresses in Strawberry (*Fragaria vesca*). Int. J. Genom..

[23] Jiang Y., Chen X., Wang F., Li X., Qin Z., Fan S., Yan N., Xie Y., Zhao R. (2025). Metabolomic response of Zizania latifolia to low-temperature stress and identification of the bZIP transcription factor family. GM Crops Food.

[24] Yang Z., Sun J., Chen Y., Zhu P., Zhang L., Wu S., Ma D., Cao Q., Li Z., Xu T. (2019). Genome-wide identification, structural and gene expression analysis of the bZIP transcription factor family in sweet potato wild relative Ipomoea trifida. BMC Genet..

[25] Banerjee A., Roychoudhury A. (2017). Abscisic-acid-dependent basic leucine zipper (bZIP) transcription factors in plant abiotic stress. Protoplasma.

[26] Amir Hossain M., Lee Y., Cho J.I., Ahn C.H., Lee S.K., Jeon J.S., Kang H., Lee C.H., An G., Park P.B. (2010). The bZIP transcription factor *OsABF1* is an ABA responsive element binding factor that enhances abiotic stress signaling in rice. Plant Mol. Biol..

[27] Basso M.F., Iovieno P., Capuana M., Contaldi F., Ieri F., Menicucci F., Celso F.L., Barone G., Martinelli F. (2025). Identification and expression of the AREB/ABF/ABI5 subfamily genes in chickpea and lentil reveal major players involved in ABA-mediated defense response to drought stress. Planta.

[28] Zou M., Guan Y., Ren H., Zhang F., Chen F. (2008). A bZIP transcription factor, OsABI5, is involved in rice fertility and stress tolerance. Plant Mol. Biol..

[29] Hossain M.A., Cho J.I., Han M., Ahn C.H., Jeon J.S., An G., Park P.B. (2010). The ABRE-binding bZIP transcription factor *OsABF2* is a positive regulator of abiotic stress and ABA signaling in rice. J. Plant Physiol..

[30] Orellana S., Yañez M., Espinoza A., Verdugo I., González E., Ruiz-Lara S., Casaretto J.A. (2010). The transcription factor *SlAREB1* confers drought, salt stress tolerance and regulates biotic and abiotic stress-related genes in tomato. Plant Cell Environ..

[31] Kim S., Kang J.Y., Cho D.I., Park J.H., Kim S.Y. (2004). ABF2, an ABRE-binding bZIP factor, is an essential component of glucose signaling and its overexpression affects multiple stress tolerance. Plant J..

[32] Kim J.H., Hyun W.Y., Nguyen H.N., Jeong C.Y., Xiong L., Hong S.W., Lee H. (2015). *AtMyb7*, a subgroup 4 R2R3 Myb, negatively regulates ABA-induced inhibition of seed germination by blocking the expression of the bZIP transcription factor ABI5. Plant Cell Environ..

[33] Skubacz A., Daszkowska-Golec A., Szarejko I. (2016). The Role and Regulation of *ABI5* (*ABA-Insensitive 5*) in Plant Development, Abiotic Stress Responses and Phytohormone Crosstalk. Front. Plant Sci..

[34] Maier A.T., Stehling-Sun S., Wollmann H., Demar M., Hong R.L., Haubeiss S., Weigel D., Lohmann J.U. (2009). Dual roles of the bZIP transcription factor PERIANTHIA in the control of floral architecture and homeotic gene expression. Development.

[35] Abe M., Kobayashi Y., Yamamoto S., Daimon Y., Yamaguchi A., Ikeda Y., Ichinoki H., Notaguchi M., Goto K., Araki T. (2005). FD, a bZIP protein mediating signals from the floral pathway integrator FT at the shoot apex. Science.

[36] Shu K., Chen Q., Wu Y., Liu R., Zhang H., Wang S., Tang S., Yang W., Xie Q. (2016). ABSCISIC ACID-INSENSITIVE 4 negatively regulates flowering through directly promoting Arabidopsis *FLOWERING LOCUS C* transcription. J. Exp. Bot..

[37] Howell S.H. (2013). Endoplasmic reticulum stress responses in plants. Annu. Rev. Plant Biol..

[38] Oñate L., Vicente-Carbajosa J., Lara P., Díaz I., Carbonero P. (1999). Barley *BLZ2*, a seed-specific bZIP protein that interacts with BLZ1 in vivo and activates transcription from the GCN4-like motif of B-hordein promoters in barley endosperm. J. Biol. Chem..

[39] Onodera Y., Suzuki A., Wu C.Y., Washida H., Takaiwa F. (2001). A rice functional transcriptional activator, RISBZ1, responsible for endosperm-specific expression of storage protein genes through GCN4 motif. J. Biol. Chem..

[40] Vicente-Carbajosa J., Oñate L., Lara P., Diaz I., Carbonero P. (1998). Barley *BLZ1*: A bZIP transcriptional activator that interacts with endosperm-specific gene promoters. Plant J..

[41] Mueller S., Hilbert B., Dueckershoff K., Roitsch T., Krischke M., Mueller M.J., Berger S. (2008). General detoxification and stress responses are mediated by oxidized lipids through TGA transcription factors in Arabidopsis. Plant Cell.

[42] Zander M., La Camera S., Lamotte O., Métraux J.P., Gatz C. (2010). Arabidopsis thaliana class-II TGA transcription factors are essential activators of jasmonic acid/ethylene-induced defense responses. Plant J..

[43] Jin H., Choi S.M., Kang M.J., Yun S.H., Kwon D.J., Noh Y.S., Noh B. (2018). Salicylic acid-induced transcriptional reprogramming by the HAC-NPR1-TGA histone acetyltransferase complex in Arabidopsis. Nucleic Acids Res..

[44] Hussain R.M.F., Sheikh A.H., Haider I., Quareshy M., Linthorst H.J.M. (2018). Arabidopsis WRKY50 and TGA Transcription Factors Synergistically Activate Expression of PR1. Front. Plant Sci..

[45] Ndamukong I., Abdallat A.A., Thurow C., Fode B., Zander M., Weigel R., Gatz C. (2007). SA-inducible Arabidopsis glutaredoxin interacts with TGA factors and suppresses JA-responsive PDF1.2 transcription. Plant J..

[46] Gachon F., Olela F.F., Schaad O., Descombes P., Schibler U. (2006). The circadian PAR-domain basic leucine zipper transcription factors DBP, TEF, and HLF modulate basal and inducible xenobiotic detoxification. Cell Metab..

[47] Gibalová A., Reňák D., Matczuk K., Dupl’áková N., Cháb D., Twell D., Honys D. (2009). *AtbZIP34* is required for Arabidopsis pollen wall patterning and the control of several metabolic pathways in developing pollen. Plant Mol. Biol..

[48] Assunção A.G., Herrero E., Lin Y.F., Huettel B., Talukdar S., Smaczniak C., Immink R.G., van Eldik M., Fiers M., Schat H. (2010). *Arabidopsis thaliana* transcription factors *bZIP19* and *bZIP23* regulate the adaptation to zinc deficiency. Proc. Natl. Acad. Sci. USA.

[49] Inaba S., Kurata R., Kobayashi M., Yamagishi Y., Mori I., Ogata Y., Fukao Y. (2015). Identification of putative target genes of *bZIP19*, a transcription factor essential for Arabidopsis adaptation to Zn deficiency in roots. Plant J..

[50] Lopes-da-Silva M., McCormack J.J., Burden J.J., Harrison-Lavoie K.J., Ferraro F., Cutler D.F. (2019). A GBF1-Dependent Mechanism for Environmentally Responsive Regulation of ER-Golgi Transport. Dev. Cell.

[51] Giri M.K., Singh N., Banday Z.Z., Singh V., Ram H., Singh D., Chattopadhyay S., Nandi A.K. (2017). *GBF1* differentially regulates *CAT2* and *PAD4* transcription to promote pathogen defense in Arabidopsis thaliana. Plant J..

[52] Yang Y., Liang T., Zhang L., Shao K., Gu X., Shang R., Shi N., Li X., Zhang P., Liu H. (2018). UVR8 interacts with WRKY36 to regulate *HY5* transcription and hypocotyl elongation in Arabidopsis. Nat. Plants.

[53] Osterlund M.T., Hardtke C.S., Wei N., Deng X.W. (2000). Targeted destabilization of *HY5* during light-regulated development of Arabidopsis. Nature.

[54] Li J., Terzaghi W., Gong Y., Li C., Ling J.J., Fan Y., Qin N., Gong X., Zhu D., Deng X.W. (2020). Modulation of BIN2 kinase activity by HY5 controls hypocotyl elongation in the light. Nat. Commun..

[55] Tsugama D., Liu S., Takano T. (2014). Analysis of functions of VIP1 and its close homologs in osmosensory responses of *Arabidopsis thaliana*. PLoS ONE.

[56] Tsugama D., Liu S., Fujino K., Takano T. (2018). Calcium signalling regulates the functions of the bZIP protein *VIP1* in touch responses in Arabidopsis thaliana. Ann. Bot..

[57] Choi H., Hong J., Ha J., Kang J., Kim S.Y. (2000). *ABFs,* a family of ABA-responsive element binding factors. J. Biol. Chem..

[58] Fukazawa J., Sakai T., Ishida S., Yamaguchi I., Kamiya Y., Takahashi Y. (2000). Repression of shoot growth, a bZIP transcriptional activator, regulates cell elongation by controlling the level of gibberellins. Plant Cell.

[59] Izawa T., Foster R., Nakajima M., Shimamoto K., Chua N.H. (1994). The rice bZIP transcriptional activator *RITA-1* is highly expressed during seed development. Plant Cell.

[60] An P., Li X., Liu T., Shui Z., Chen M., Gao X., Wang Z. (2022). The Identification of Broomcorn Millet bZIP Transcription Factors, Which Regulate Growth and Development to Enhance Stress Tolerance and Seed Germination. Int. J. Mol. Sci..

[61] Chen C., Chen H., Zhang Y., Thomas H.R., Frank M.H., He Y., Xia R. (2020). TBtools: An Integrative Toolkit Developed for Interactive Analyses of Big Biological Data. Mol. Plant.

[62] Liu Z., Zhang M., Kong L., Lv Y., Zou M., Lu G., Cao J., Yu X. (2014). Genome-wide identification, phylogeny, duplication, and expression analyses of two-component system genes in Chinese cabbage (*Brassica rapa* ssp. pekinensis). DNA Res. Int. J. Rapid Publ. Rep. Genes. Genomes.

[63] Krishnamurthy P., Hong J.K., Kim J.A., Jeong M.-J., Lee Y.-H., Lee S.I. (2015). Genome-wide analysis of the expansin gene superfamily reveals Brassica rapa-specific evolutionary dynamics upon whole genome triplication. Mol. Genet. Genom. MGG.

[64] Barker M.S., Baute G.J., Liu S.L. (2012). Duplications and Turnover in Plant Genomes. Plant Genome Diversity.

[65] Zhang J. (2003). Evolution by gene duplication: An update. Trends Ecol. Evol..

[66] Jacob A.G., Smith C.W.J. (2017). Intron retention as a component of regulated gene expression programs. Hum. Genet..

[67] Wang W., Wang Y., Zhang S., Xie K., Zhang C., Xi Y., Sun F. (2020). Genome-wide analysis of the abiotic stress-related *bZIP* family in switchgrass. Mol. Biol. Rep..

[68] Hu W., Wang L., Tie W., Yan Y., Ding Z., Liu J., Li M., Peng M., Xu B., Jin Z. (2016). Genome-wide analyses of the *bZIP* family reveal their involvement in the development, ripening and abiotic stress response in banana. Sci. Rep..

[69] Yang S., Xu K., Chen S., Li T., Xia H., Chen L., Liu H., Luo L. (2019). A stress-responsive bZIP transcription factor *OsbZIP62* improves drought and oxidative tolerance in rice. BMC Plant Biol..

[70] Ma H., Liu C., Li Z., Ran Q., Xie G., Wang B., Fang S., Chu J., Zhang J. (2018). *ZmbZIP4* Contributes to Stress Resistance in Maize by Regulating ABA Synthesis and Root Development. Plant Physiol..

[71] Zhang M., Liu Y., Cai H., Guo M., Chai M., She Z., Ye L., Cheng Y., Wang B., Qin Y. (2020). The bZIP Transcription Factor *GmbZIP15* Negatively Regulates Salt- and Drought-Stress Responses in Soybean. Int. J. Mol. Sci..

[72] Bi C., Yu Y., Dong C., Yang Y., Zhai Y., Du F., Xia C., Ni Z., Kong X., Zhang L. (2021). The bZIP transcription factor *TabZIP15* improves salt stress tolerance in wheat. Plant Biotechnol. J..

[73] Zhao P., Ye M., Wang R., Wang D., Chen Q. (2020). Systematic identification and functional analysis of potato (*Solanum tuberosum* L.) bZIP transcription factors and overexpression of potato bZIP transcription factor *StbZIP-65* enhances salt tolerance. Int. J. Biol. Macromol..

[74] He Q., Cai H., Bai M., Zhang M., Chen F., Huang Y., Priyadarshani S.V.G.N., Chai M., Liu L., Liu Y. (2020). A Soybean bZIP Transcription Factor Confers Multiple Biotic and Abiotic Stress Responses in Plant. Int. J. Mol. Sci..

[75] Cai W., Yang Y., Wang W., Guo G., Liu W., Bi C. (2018). Overexpression of a wheat (*Triticum aestivum* L.) bZIP transcription factor gene, *TabZIP6*, decreased the freezing tolerance of transgenic Arabidopsis seedlings by down-regulating the expression of CBFs. Plant Physiol. Biochem..

