# Supplementary material for: Genome-Wide Identification and Analysis of bZIP Transcription Factor Gene Family in Broomcorn Millet (Panicum miliaceum L.)
Source: Genes (Basel). 2025 Jun 24;16(7):734. doi: 10.3390/genes16070734 (PMC12295044; doi:10.3390/genes16070734)
Supplement: Supplementary file 1 [file genes-16-00734-s001.zip › genes-3714200-supplementary.pdf]

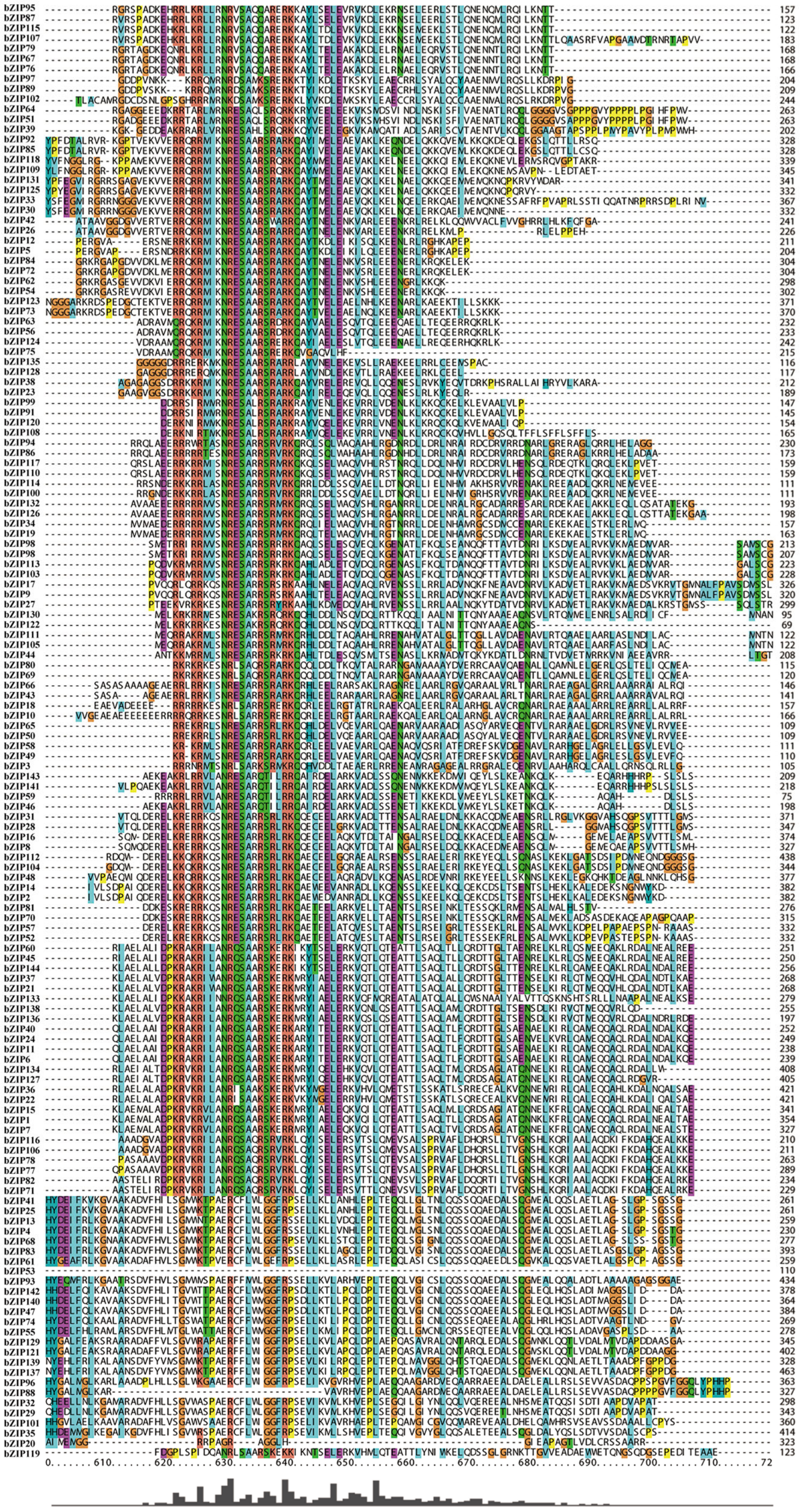

Figure S1. Sequence conservation analysis of PmbZIPs.

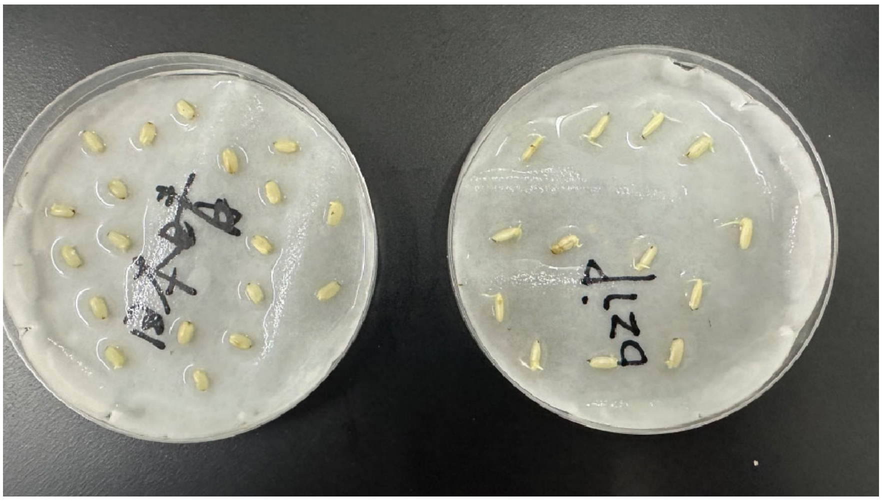

Figure S2. Overexpression of *PmbZIP30* in rice accelerates seed germination. Germination under water was compared between Nipponbare (wild-type) and *PmbZIP30*-overexpressing rice lines. It was observed that the *PmbZIP30* transgenic lines germinated significantly earlier than the Nipponbare, with germination initiation occurring at 12 hours post-imbibition in transgenic lines versus 24-36 hours in Nipponbare.
